# Is older age associated with COVID-19 mortality in the absence of other risk factors? General population cohort study of 470,034 participants

**DOI:** 10.1371/journal.pone.0241824

**Published:** 2020-11-05

**Authors:** Frederick K. Ho, Fanny Petermann-Rocha, Stuart R. Gray, Bhautesh D. Jani, S. Vittal Katikireddi, Claire L. Niedzwiedz, Hamish Foster, Claire E. Hastie, Daniel F. Mackay, Jason M. R. Gill, Catherine O'Donnell, Paul Welsh, Frances Mair, Naveed Sattar, Carlos A. Celis-Morales, Jill P. Pell

**Affiliations:** 1 Institute of Health and Wellbeing, University of Glasgow, Glasgow, United Kingdom; 2 Institute of Cardiovascular and Medical Sciences, University of Glasgow, Glasgow, United Kingdom; Beijing Key Laboratory of Diabetes Prevention and Research, CHINA

## Abstract

**Introduction:**

Older people have been reported to be at higher risk of COVID-19 mortality. This study explored the factors mediating this association and whether older age was associated with increased mortality risk in the absence of other risk factors.

**Methods:**

In UK Biobank, a population cohort study, baseline data were linked to COVID-19 deaths. Poisson regression was used to study the association between current age and COVID-19 mortality.

**Results:**

Among eligible participants, 438 (0.09%) died of COVID-19. Current age was associated exponentially with COVID-19 mortality. Overall, participants aged ≥75 years were at 13-fold (95% CI 9.13–17.85) mortality risk compared with those <65 years. Low forced expiratory volume in 1 second, high systolic blood pressure, low handgrip strength, and multiple long-term conditions were significant mediators, and collectively explained 39.3% of their excess risk. The associations between these risk factors and COVID-19 mortality were stronger among older participants. Participants aged ≥75 without additional risk factors were at 4-fold risk (95% CI 1.57–9.96, P = 0.004) compared with all participants aged <65 years.

**Conclusions:**

Higher COVID-19 mortality among older adults was partially explained by other risk factors. ‘Healthy’ older adults were at much lower risk. Nonetheless, older age was an independent risk factor for COVID-19 mortality.

## Introduction

COVID-19 is an emerging infectious disease caused by the novel coronavirus SARS-CoV-2 and has a wide spectrum of manifestations ranging from asymptomatic infection to severe pneumonia and respiratory failure. As of mid-August 2020, the COVID-19 pandemic has infected over 20 million people globally and caused at least 750,000 deaths [1].

Preliminary reports have shown that older people were at a higher risk of COVID-19 complications with higher rates of hospitalisation, intensive care unit admissions, intubation, and death [2–4]. Currently, it is unclear whether chronological age per se is an independent risk factor for severe COVID-19, or simply that risk factors are more common among older adults. Also, the mechanisms through which older age may predispose to poorer prognosis have yet to be elucidated. Several hypotheses have been proposed as to why older people might be more susceptible to severe COVID-19 infection, including a weaker immune response [5], obesity [6], age-related decline in respiratory function [6], frailty [7], and multimorbidity [8, 9].

These questions cannot be answered using hospital studies due to selection biases in testing and admission, nor using administrative databases because of insufficient information on confounding and mediating factors. Therefore, we used UK Biobank, a large, general population cohort study with rich pre-infection data, to identify factors that help explain the association between age and COVID-19 mortality and determine whether age per se is a risk.

## Methods

UK Biobank recruited over 502,000 participants aged 37 to 73 years (47 to 85 years as of 1 March 2020) at 22 assessment centres across England, Scotland, and Wales between March 2006 and December 2010. We excluded all participants known to have died prior to 1 March 2020, before the COVID-19 pandemic reached the UK.

UK Biobank received ethical approval from the North West Multi-Centre Research Ethics Committee (REC reference: 11/NW/03820). All participants gave written informed consent before enrolment in the study, which was conducted in accord with the principles of the Declaration of Helsinki.

### Outcomes

COVID-19 death records were based on death certificates, available on all participants up to 30 June 2020. COVID-19-related deaths were defined as ICD-10 codes U07.1 or U07.2 on the death certificates.

### Exposures

At baseline assessment, biological measurements were taken, and data were collected via both a self-administered touch-screen questionnaire and research nurse led interview according to a standardised protocol at a median 11.1 (interqartile range 10.4–11.8) years before 1 March 2020. Current age (on 1 March 2020) was derived from date of, and age at, recruitment and was trichotomised into <65, 65–74 and 75 years. Ethnicity, smoking, medical history and medication use were self-reported at baseline. For the present analyses, ethnicity was classified as white or other, due to insufficient participants in the non-white groups (n = 25,186, <6%). Smoking status was categorised into current/former smoker and never smoker. Systolic blood pressure (SBP) were measured at the baseline assessment using automated measurements (or manual if unavailable), and the mean of available measurements derived. Area-level socioeconomic deprivation was based on the Townsend score of the participant’s home postcode derived from Census data on: unemployment, non-car ownership, non-home ownership and household overcrowding. Higher Townsend scores represent greater socioeconomic deprivation [10].

Body mass index (BMI) was derived from measured body mass in kilograms divided by height squared, measured in metres. Height was measured, without shoes and socks, using a Seca 202 height measure. Weight and whole-body fat mass and fat free mass were measured to the nearest 0.1 kg using the Tanita BC-418 MA body composition analyser.

Lung function was assessed by spirometry using a Vitalograph Pneumotrac 6800 spirometer (Vitalograph, Buckingham, UK). Participants did not perform spirometry if they were pregnant, on medication for tuberculosis or had a history of: chest infection (in the last month); detached retina; myocardial infarction; eye, chest or abdominal surgery (in the last three months); or collapsed lung. The aim was to record two acceptable blows from a maximum of three attempts. The spirometer software compared the acceptability of the first two blows and, if acceptable (defined as ≤5% difference in FVC and FEV_1_), the third blow was not required. In the moderation analyses, we used the height-, sex-, and ethnicity-specific predicted FEV_1_ value at 65 years of age from the Global Lung Function Initiative (GLI) [11] as the cut-off value to define normal versus low FEV_1_, because participants who were 75 years of age during the pandemic were around 65 years of age at baseline.

The Fried classification uses five criteria: weight loss, exhaustion, physical inactivity, slow walking speed and low grip strength. Grip strength was measured using a Jamar J00105 hydraulic hand dynamometer and the mean was derived from the right and left hand values expressed in kilograms. Self-reported walking pace was categorised as slow, average, or brisk. An adapted version of the frailty classification derived by Fried et al. was used in this study [12]. Participants were classified as frail if they fulfilled three or more criteria, prefrail if they fulfilled one or two criteria and robust (non-frail) if they did not fulfil any criteria.

The information collected on long-term conditions (LTCs) during the nurse-led interview (full list contained in S1 Table) was converted into the total number of LTCs for each participant.

### Statistical analyses

Means and standard deviations were reported for continuous variables and numbers and percentages for categorical variables. Poisson regression models with robust standard errors were used to analyse the associations between risk factors and COVID-19 mortality, with the results reported as risk ratios (RRs) and 95% confidence intervals (CIs) [13]. Poisson regression models were used instead of logistic regression because they provide RR estimates which aid clinical interpretation.

We used penalised thin plate regression splines to model the association between age and COVID-19 mortality as it may not be linear [14]. Splines were chosen over fractional polynomials in capturing deep curvatures [15]. Penalised thin plate regression splines provide more robust results than cubic splines as knot locations do not need to be chosen [16].

The main analyses were adjusted for potential confounding factors: sex, ethnic group, deprivation index and smoking status. We studied four groups of potential mediators: physical (BMI, SBP), respiratory (FEV_1_ and FEV_1_ / FVC ratio), frailty (non-frail, prefrail and frail), and number of LTCs. These factors were included as covariates in the Poisson models to determine whether, and to what extent, the RRs between age and COVID-19 were attenuated. In addition, mediation analysis under counterfactual framework was also conducted [17]. To avoid multicollinearity and unnecessary adjustment between potential mediators, potential mediators were selected in a stepwise process. Firstly, COVID-19 was regressed by current age, all potential mediators, and confounding factors in a Poisson model. Only the potential mediators reaching statistical significance (α = 0.05) were further investigated. Factors that were of high effect sizes (RR <0.9 or RR >1.1) were also considered. The selected potential mediators were then regressed by age and other covariates (mediator model) in either multiple Poisson (for binary mediators) or linear (for other mediators) models adjusting for each other and for sociodemographic factors. The outcome and mediator models were then combined to compute the natural indirect effect (NIE) and total effect (TE) for each participant which was then averaged. Quasi-Bayesian estimation with 1,000 iterations were used for estimating the 95% CI and p-values of the NIE and TE. Mediation proportion was calculated as NIE / TE.

The potential moderating role of risk factors in the association between age and COVID-19 mortality was studied in a series of subgroup analyses using combinations of age (<64, 65–74, and ≥75 years) and risk factors: current/former smokers, low FEV_1_ (< height-, sex-, and ethnicity predicted value at 65 years old [11]), obesity (BMI >30 kg/m^2^), hypertension (SBP ≥140 mmHg, DBP ≥90 mmHG or antihypertensive medication), frailty (prefrail and frail), and ≥3 LTCs. These variables were categorised for easier interpretation. The interactions between risk factors and age group were tested using likelihood ratio tests comparing models with and without the interaction terms. Each risk factor was combined with age group and the RR derived for each permutation referent to participants aged <65 years of age and without the risk factor. This was repeated for the combinations of age and total number of risk factors.

Missing data were handled using complete case analysis. All analyses were conducted using R version 4.0.2 with package *mgcv* and *mediation*.

## Results

Of the 502,506 UK Biobank participants, we excluded 29,295 who died prior to 1 March 2020 and 3,177 who had incomplete data on potential confounding factors, resulting in 470,034 participants being included in the analyses of COVID-19 mortality (S1 Fig). Overall, 438 participants died of COVID-19.

Older particpants were less deprived, less likely to be current smokers, more likely to be frail, and had higher SBP, lower handgrip strength, poorer lung function, and more LTCs (Table 1). Participants who died of COVID-19 were older, less likely to be white, more likely to smoke, be male, obese and frail, and had more LTCs, higher SBP and poorer lung function (Table 1).

**Table 1:** Participant characteristics.

	Current age (years)			COVID-19 mortality	
	<65 N = 179,249	65–74 N = 196,290	≥75 N = 94,495	Alive N = 469,596	Died N = 438
Mean (SD) current age, years	57.95 (4.04)	69.87 (2.79)	77.17 (1.74)	66.78 (8.09)	73.43 (5.90)
Mean (SD) age at baseline, years	47.42 (4.06)	59.33 (2.96)	66.48 (1.77)	56.22 (8.08)	62.81 (5.81)
Male	79135 (44.15)	86193 (43.91)	44691 (47.29)	209744 (44.66)	275 (62.79)
Ethnic minority	15855 (8.85)	7450 (3.80)	2663 (2.82)	25930 (5.52)	38 (8.68)
Mean (SD) deprivation index	-0.97 (3.21)	-1.53 (2.96)	-1.65 (2.91)	-1.34 (3.06)	-0.13 (3.48)
Smoking status					
Never	108694 (60.80)	105025 (53.70)	47597 (50.65)	261150 (55.81)	166 (38.07)
Previous	46870 (26.22)	73076 (37.36)	40157 (42.73)	159899 (34.17)	204 (46.79)
Current	23212 (12.98)	17473 (8.93)	6224 (6.62)	46843 (10.01)	66 (15.14)
Mean (SD) BMI, kg/m^2^	27.12 (4.85)	27.53 (4.67)	27.51 (4.29)	27.37 (4.67)	29.34 (5.52)
BMI categories					
Underweight	1065 (0.60)	884 (0.45)	369 (0.39)	2315 (0.50)	3 (0.70)
Normal	65202 (36.55)	61309 (31.36)	27297 (29.02)	153724 (32.88)	84 (19.58)
Overweight	70988 (39.80)	84306 (43.13)	43881 (46.65)	198994 (42.57)	181 (42.19)
Obese	41126 (23.06)	48973 (25.05)	22527 (23.95)	112465 (24.06)	161 (37.53)
Mean (SD) SBP, mmHg	130.93 (16.33)	139.74 (18.00)	145.54 (18.49)	137.53 (18.36)	144.99 (18.89)
Mean (SD) handgrip strength, kg	32.83 (11.05)	29.77 (10.80)	28.31 (10.32)	30.65 (10.95)	29.77 (10.35)
Mean (SD) FEV_1_, L	3.11 (0.77)	2.74 (0.72)	2.50 (0.69)	2.84 (0.77)	2.52 (0.84)
Mean (SD) FEV_1_/FVC	0.77 (0.06)	0.75 (0.06)	0.74 (0.07)	0.76 (0.06)	0.73 (0.08)
Frailty stages					
Non-frail	71093 (51.13)	73334 (48.27)	34384 (46.31)	178687 (48.97)	124 (40.00)
Pre-frail	63825 (45.91)	72785 (47.91)	36840 (49.62)	173288 (47.49)	162 (52.26)
Frail	4113 (2.96)	5811 (3.82)	3027 (4.08)	12927 (3.54)	24 (7.74)
Number of LTCs					
0	83896 (46.80)	62178 (31.68)	20861 (22.08)	166858 (35.53)	77 (17.58)
1	57883 (32.29)	66375 (33.81)	30726 (32.52)	154870 (32.98)	114 (26.03)
2	24524 (13.68)	39827 (20.29)	23390 (24.75)	87631 (18.66)	110 (25.11)
3	8583 (4.79)	17662 (9.00)	12141 (12.85)	38303 (8.16)	83 (18.95)
4	2897 (1.62)	6650 (3.39)	4802 (5.08)	14318 (3.05)	31 (7.08)
≥5	1466 (0.82)	3598 (1.83)	2575 (2.73)	7616 (1.62)	23 (5.25)

N number; SD standard deviation; SBP systolic blood pressure, BMI body mass index; FEV forced expiratory volume; FVC forced vital capacity; LTC long-term condition

Numbers presented are n (%) unless otherwise specified

**Fig 1:**
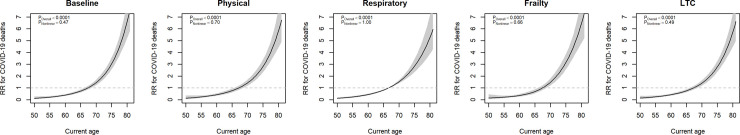
Association of age with COVID-19 mortality by adjustment schemes. Model 1 (Baseline): Sex, ethnicity, deprivation, duration of follow-up, smoking; Model 2 (Physical): Baseline + BMI, SBP; Model 3 (Respiratory): Baseline + FEV_1_, FEV1/FVC; Model 4 (Frailty): Baseline + frailty stages; Model 5 (LTC): Baseline + number of LTCs; BMI body mass index; SBP systolic blood pressure; FEV forced expiratory volume; FVC forced vital capacity; LTC long-term conditions.

Current age was associated exponentially with COVID-19 mortality (Fig 1). Adjusting for physical (Model 2), respiratory (Model 3), and LTC (Model 5) covariates attenuated the association. There was no evidence of a non-linear association between age and the logarithm of mortality risk.

After adjusting for potential confounding factors and other age-related risk factors, only BMI, SBP, handgrip strength, FEV_1_, and ≥3 LTCs were significantly associated with COVID-19 mortality. BMI was excluded in the mediation analysis as it was inversely associated with age after adjustment for other potential mediators. Therefore, mediation analysis was conducted on FEV_1_ SBP, handgrip strength, and LTCs. These factors collectively accounted for 39.3% of the association between older age and COVID-19 mortality (Table 2).

**Table 2:** Potential mediators of the association between age ≥75 years and COVID-19 mortality.

	Association with COVID-19 outcomes		Regressed by older age		Mediation	
	RR (95% CI)	P	RR / β^†^ (95% CI)	P	%	P
FEV_1_^†^	0.66 (0.56, 0.78)	<0.0001	-0.40 (-0.41, -0.39)	<0.0001	16.8	<0.0001
SBP^†^	1.25 (1.11, 1.42)	0.0002	0.40 (0.39, 0.41)	<0.0001	9.9	<0.0001
Handgrip strength^†^	0.74 (0.61, 0.89)	0.001	-0.19 (-0.19, -0.18)	<0.0001	6.5	<0.0001
LTC ≥3	1.64 (1.27, 2.13)	0.0002	1.22 (1.21, 1.24)	<0.0001	6.1	<0.0001

RR relative risk; CI confidence interval; FEV forced expiratory volumne; LTC long-term conditions

^†^Continuous variables were expressed per 1-SD

Adjusted for sex, ethnicity, deprivation, duration of follow-up, and smoking

**Fig 2:**
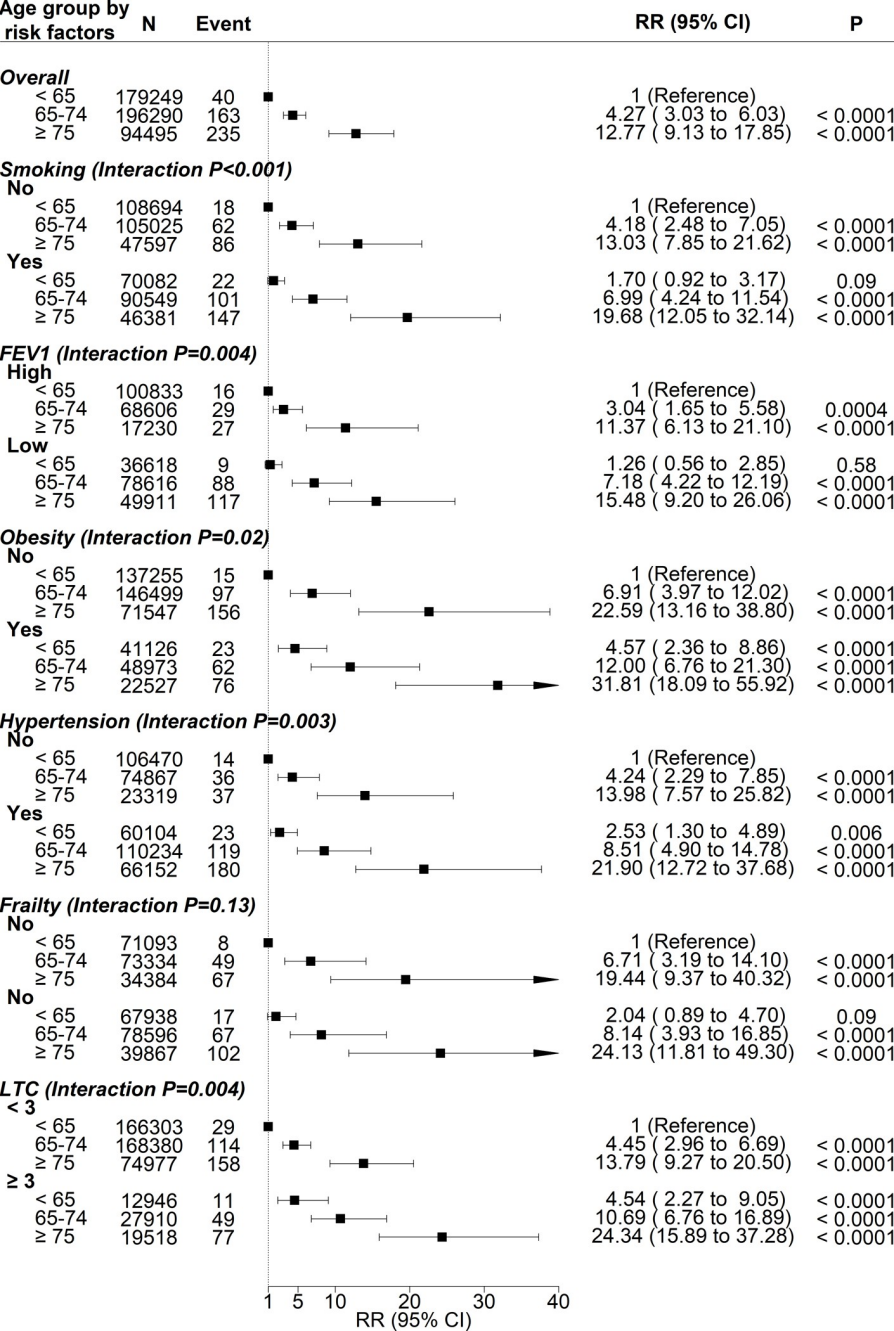
Associations between combinations of age group and risk factors and COVID-19 mortality. Adjusted for sex, ethnicity, deprivation, duration of follow-up; N number; RR relative risk; CI confidence interval; FEV forces expiratory volume; LTC long-term condition.

There were statistically significant interactions between age group and all risk factors in relation to COVID-19 mortality, except for frailty. Fig 2 shows the associations with COVID-19 mortality of different combinations of age and risk factors. Compared with participants <65 years of age who had never smoked, participants ≥75 years of age had a higher risk even if they had never smoked (RR 13.03, 95% CI 7.85–21.62, P<0.0001) and higher still if they had ever smoked (RR 19.68, 95% CI 12.05–32.14, P<0.0001). A similar pattern was observed for FEV_1_, obesity, hypertension, and number of LTCs.

**Fig 3:**
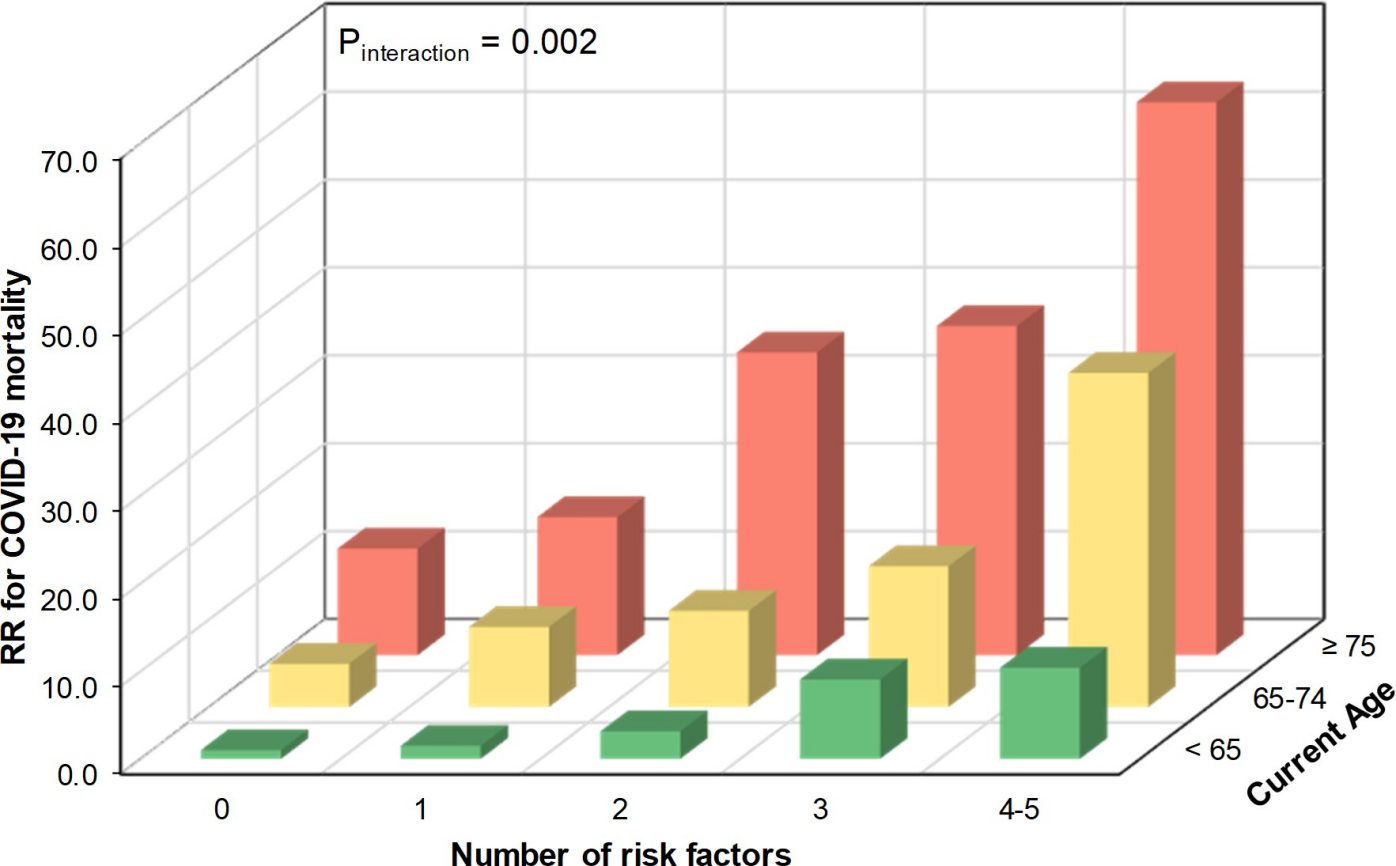
Association between age group combined with number of risk factors and COVID-19 mortality. Adjusted for sex, ethnicity, deprivation, duration of follow-up Risk factors included smoking, obesity, hypertension, FEV_1_, frailty, and number of LTCs ≥3.

Fig 3 shows the associations between age group and number of risk factors with COVID-19 mortality. Overall, participants aged ≥75 years were at 13-fold (95% CI 9.13–17.85) mortality risk compared with those <65 years. The association between number of risk factors and COVID-19 mortality was stronger among older participants. Participants aged ≥75 years with no additional risk factors (smoking, low FEV_1_, obesity, hypertension, frailty, and multiple LTCs) had 12-fold mortality risk (RR 12.13, 95% CI 2.79–52.66, P = 0.0009) compared with those aged <65 years with no risk factors, and had 4-fold mortality risk (95% CI 1.57–9.96, P = 0.004) compared with all participants aged <65 years (S2 Table).

## Discussion

### Principal findings

This study demonstrated an exponential association between age and COVID-19 mortality. Over one-third of older adults’ excess mortality risk was mediated by poorer lung function, hypertension, muscle weakness, and multiple LTCs. Among older participants, these factors were both more common and more strongly associated with higher COVID-19 mortality.

FEV_1_ is a commonly used marker of respiratory function [18, 19]. It is used to diagnose chronic obstructive pulmonary disease (COPD) but is also associated with mortality independent of clinical disease [18]. FEV_1_ generally peaks in early adulthood and declines with age beyond 30 to 40 years of age [19]. However, there is a large variation in the peak value and age-related rate of decline due, in part, to lifestyle factors such as smoking, obesity, and physical activity [20]. The mechanism underlying the relationship between FEV_1_ and COVID-19 merits further study but may be due to people with poorer FEV_1_ having less cardiorespiratory reserve to buffer against the immune-mediated lung response to COVID-19 infection [6].

LTCs are more common among older adults and have been shown to be associated with poorer functional health [21] and poorer outcomes in COVID-19 [22, 23]. This is consistent with other infectious diseases [24–26]. The association between LTCs and increased risk of COVID-19, as in other infectious diseases, could be related to shared biological pathways such as chronic low-grade inflammation [27, 28] and attenuated immune response [29].

### Strengths and limitations

This study used a large, general population cohort that provided extensive pre-infection data on sociodemographic factors, physical measurements, LTCs, and respiratory function. Therefore, we were able to take account of multiple confounders, identify potential mediators and undertake sub-group analyses.

However, there are several limitations to this study. COVID-related deaths relied on records on death certificates and it is possible that a small number of participants who died of COVID-19 were miscoded. However, as we have included both confirmed (ICD-10 U07.1) and suspected (ICD-10 U07.2) cases, such misclassification should be minimal. All analysed risk factors, excluding age, were assessed 10 years prior to the COVID-19 pandemic and may have changed over time. Any deterioration in these factors over time is likely to be greater in older age-groups and, therefore, the findings are biased towards the null. No participants in UK Biobank are currently aged >85 years and therefore our findings should not be generalised to people over 85 years of age. The UK Biobank cohort is not completely representative of the general UK population [30, 31]. However, effect sizes, such as risk ratios reported in this study, are still generalisable [32]. As with other observational studies, residual confounding may exist. The mediation analyses conducted assumed no causal relationship between mediators and thus could not detect sequential mediation.

### Comparison with existing studies

The majority of studies on ageing and COVID-19 have been based on hospital samples and focused on complications or case fatality. It was reported that those who required admission to intensive care units were on average 15 years older and more likely to have underlying comorbidities [33]. A recent meta-analyses of 33 studies conducted on a total of 3,027 patients with COVID-19 showed that adults older than 65 years were five times more likely to become critical or die [23]. In the US, it was estimated that COVID-19 related hospitalisation was lowest among 0–17 year-olds and increased almost linearly with age from 2.5 per 100,000 among people 18–49 years to 17.2 per 100,000 among those ≥85 years [2]. This is in contrast to our present findings that people aged below 70 shared similar risk. The inconsistency may be due to the lower test rate in the UK where tests have, so far, been largely confined to people with more severe symptoms or to the fact that the minimum current age in UK Biobank was 50 years.

## Conclusions

Our findings suggest that the risk of COVID-19 mortality is higher in older adults. In this cohort, over one-third of this excess was due to older adults being more likely to have other risk factors (e.g. poorer lung function and hypertension) and these risk factors conveying a stronger risk of COVID-19 death among older people. Nonetheless, older age was associated with COVID-19 mortality independent of other risk factors.

Currently, everyone over 70 years of age is classified as being at moderate risk from COVID-19 irrespective of their general health [34]. As such they are recommended to be more stringent in following social distancing. Our study findings suggest that efforts to protect older people should prioritise those who have additional risk factors.

## Supporting information

S1 Fig Participant flow chart. (DOCX)

S1 Table Long-term conditions recorded at baseline assessment. (DOCX)

S2 Table Association between number of risk factors and COVID-19 mortality. (DOCX)
