# Supplementary material for: Is older age associated with COVID-19 mortality in the absence of other risk factors? General population cohort study of 470,034 participants
Source: PLoS One. 2020 Nov 5;15(11):e0241824. doi: 10.1371/journal.pone.0241824 (PMC7644030; doi:10.1371/journal.pone.0241824)
Supplement: S1 Fig — (DOCX) [file pone.0241824.s001.docx]

**S1 Fig. Participant flow chart**

All UK Biobank participants

N = 502,506

Participants alive up to 1 March 2020

N = 473,211

29,295 particpiants died before 1 March 2020 excluded

Participants analysed

N = 470,034

3,177 particpiants without complete sociodemographic data excluded
