# Supplementary material for: Is older age associated with COVID-19 mortality in the absence of other risk factors? General population cohort study of 470,034 participants
Source: PLoS One. 2020 Nov 5;15(11):e0241824. doi: 10.1371/journal.pone.0241824 (PMC7644030; doi:10.1371/journal.pone.0241824)
Supplement: S1 Table — (DOCX) [file pone.0241824.s002.docx]

**S1 Table. Long-term conditions recorded at baseline assessment**

| 1. Hypertension 2. Depression 3. Painful condition 4. Asthma 5. Ischaemic heart disease 6. Dyspepsia 7. Diabetes 8. Thyroid 9. Inflammation (rheumatoid arthritis/ other inflammation) 10. Chronic obstructive pulmonary disease 11. Anxiety 12. Irritable bowel syndrome 13. Alcohol problems 14. Other psychoactive substance abuse 15. Constipation 16. Stroke 17. Chronic Kidney disease 18. Diverticular disease of intestine 19. Atrial fibrillation 20. Peripheral vascular disease 21. Heart failure 22. Prostate disorders 23. Glaucoma 24. Epilepsy 25. Dementia 26. Psoriasis or eczema 27. Inflammatory bowel disease 28. Migraine 29. Chronic sinusitis 30. Anorexia or bulimia 31. Bronchiectasis 32. Parkinson’s disease 33. Multiple sclerosis 34. Chronic liver disease 35. Osteoporosis 36. Chronic fatigue syndrome 37. Endometriosis 38. Polycystic ovary syndrome 39. Pernicious anaemia 40. Cancer |
| --- |
