# Supplementary material for: Is older age associated with COVID-19 mortality in the absence of other risk factors? General population cohort study of 470,034 participants
Source: PLoS One. 2020 Nov 5;15(11):e0241824. doi: 10.1371/journal.pone.0241824 (PMC7644030; doi:10.1371/journal.pone.0241824)
Supplement: S2 Table — (DOCX) [file pone.0241824.s003.docx]

**S2 Table. Association between number of risk factors and COVID-19 mortality**

|  | **RR (95% CI)** | **P** |
| --- | --- | --- |
| **All <65 years** | 1 (Reference) |  |
| **65-74 years** |  |  |
| 0 | 1.60 ( 0.75, 3.41) | 0.22 |
| 1 | 2.96 ( 1.68, 5.21) | 0.0002 |
| 2 | 3.54 ( 2.06, 6.09) | < 0.0001 |
| 3 | 5.17 ( 2.94, 9.06) | < 0.0001 |
| 4-5 | 12.19 ( 7.21, 20.60) | < 0.0001 |
| **≥75 years** |  |  |
| 0 | 3.95 ( 1.57, 9.96) | 0.004 |
| 1 | 5.11 ( 2.69, 9.73) | < 0.0001 |
| 2 | 11.18 ( 7.03, 17.80) | < 0.0001 |
| 3 | 12.11 ( 7.41, 19.80) | < 0.0001 |
| 4-5 | 20.22 (12.36, 33.07) | < 0.0001 |

RR relative risk; CI confidence interval

Adjusted for sex, ethnicity, deprivation, duration of follow-up, and smoking
